# High concordance in preimplantation genetic testing for aneuploidy between automatic identification via Ion S5 and manual identification via Miseq

**DOI:** 10.1038/s41598-021-98318-9

**Published:** 2021-09-23

**Authors:** Tzu-Hsuan Chuang, Zih-Huei Wu, Chin-Sheng Kuan, Meng-Ju Lee, Chia-Lin Hsieh, Huai-Lin Wang, Hsing-Hua Lai, Yu-Jen Chang, Shee-Uan Chen

**Affiliations:** 1 Stork Fertility Center, Stork Ladies Clinic, Hsinchu, Taiwan; 2 Graduate Institute of Clinical Medicine, National Taiwan University and College of Medicine, Taipei, Taiwan; 3 Bioresource Collection and Research Center, Food Industry Research and Development Institute, Hsinchu, Taiwan; 4 Department of Obstetrics and Gynecology, National Taiwan University Hospital and College of Medicine, No. 8, Chung-Shan South Road, Taipei, Taiwan

**Keywords:** Genomic analysis, High-throughput screening, Molecular medicine

## Abstract

The Ion S5 (Thermo Fisher Scientific) and Miseq (Illumina) NGS systems are both widely used in the clinical laboratories conducting PGT-A. Each system employs discrepant library preparation steps, sequencing principles, and data processing algorithms. The automatic interpretation via Ion Reporter software (Thermo Fisher Scientific) and the manual interpretation via BlueFuse Multi software (Illumina) for chromosomal copy number variation (CNV) represent very different reporting approaches. Thus, it is intriguing to compare their ability of ploidy detection as PGT-A/NGS system. In the present study, four aneuploid cell lines were individually mixed with a diploid cell line at different aneuploid ratios of 0% (0:5), 10% (1:9), 20% (1:4), 40% (2:3), 50% (3:3), 60% (3:2), 80% (4:1) and 100% (5:0) to assess the sensitivity and specificity for whole chromosomal and segmental aneuploidy detection. The clinical biopsies of 107 blastocysts from 46 IVF/PGT-A cycles recruited between December 2019 and February 2020 were used to calculate the concordance. Initially, the pre-amplified products were divided into two aliquots for different library preparation procedures of each system. Applying the same calling criteria, automatic identification was achieved through the Ion Reporter, while well-trained technicians manually identified each sample through the BlueFuse Multi. The results displayed that both systems reliably distinguished chromosomal CNV of the mixtures with at least 10% aneuploidy from karyotypically normal samples ([Ion S5] whole-chromosomal duplication: 2.14 vs. 2.05, *p* value = 0.009, segmental deletion: 1.88 vs. 2.05, *p* value = 0.003; [Miseq] whole-chromosomal duplication: 2.12 vs. 2.03, *p* value = 0.047, segmental deletion: 1.82 vs. 2.03, *p* value = 0.002). The sensitivity and specificity were comparable between the Ion S5 and Miseq ([sensitivity] 93% vs. 90%, *p* = 0.78; [specificity] 100% vs. 100%, *p* value = 1.0). In the 107 clinical biopsies, three displayed chaotic patterns (2.8%), which could not be interpreted for the ploidy. The ploidy concordance was 99.04% (103/104) per embryo and 99.47% (2265/2277) per chromosome pair. Since their ability of detection were proven to be similar, the automatic identification in Ion S5 system presents comparatively faster and more standardized performance.

## Introduction

Preimplantation genetic testing for aneuploidy (PGT-A) was developed to detect imbalanced chromosome number in the early-stage embryos during IVF and has been continuously improved. It is utilized to identify chromosomal copy number variation (CNV) in a single biopsy of embryos via different comprehensive chromosome screening (CCS) tools^1^. Initially, the fluorescence in situ hybridization (FISH) technique was employed, but it led to poor clinical outcomes as only a few chromosome pairs could be detected^2^. Following the emergence of array comparative genomic hybridization (aCGH), it soon became widely used for 24-chromosome CNV analysis^3^. Simultaneously, trophectoderm (TE) biopsy of blastocysts was gradually developed, and shown to overcome the mosaicism issue in the biopsy of day 3 cleavage-stage embryos^4,5^. Therefore, analysis of TE biopsies using aCGH was commonly accepted by laboratories conducting PGT-A. In addition to aCGH, quantitative polymerase chain reaction (qPCR) and single nucleotide polymorphism microarrays (SNP arrays) have also been demonstrated as CCS methodologies for embryo ploidy^6–8^.

In 2013, next-generation sequencing (NGS) for chromosomal CNV analysis was introduced in PGT-A^9^. It offers the advantages of high throughput and increased flexibility of data analysis. Therefore, it efficiently reduced costs and enhanced sensitivity^10^. In order to analyze samples containing only 5 to 10 cells, the biopsies must undergo whole-genome amplification (WGA) initially. Then, the amplified DNAs are pooled to create a library for massive sequencing. Within the massive sequencing, the number of reads generated determines how much information can be obtained from each individual sample and how many samples can be simultaneously tested in a single run^11^. To calculate CNV, each chromosome is divided into several intervals of appropriate unit lengths, so-called as ‘bins’ or ‘tiles,’ and the reads that pass quality assurance metrics are mapped to the human reference genome according to the intervals. Then, the bin count data is calculated, corrected, and smoothed using commercialized algorithms specific to different interpretation software. The chromosomal CNV can be distinguished by the deviation of default copy number representing as two^10^.

Apart from detecting whole chromosome aneuploidy, NGS technologies can also identify segmental or mosaic aneuploidies^12–14^. Based on validation against karyotypically defined samples, NGS was proven to be able to detect the above aneuploidies, though the sensitivity and specificity were highly dependent on the calling conditions^15^. In addition to the applied criteria, technical derivatives due to WGA artifacts and masking effects conferred by the algorithms can also be the source of bias that reduces the accuracy of PGT-A^16,17^.

The Ion S5 system (Thermo Fisher Scientific, Waltham, MA, USA) and Miseq system (Illumina, San Diego, CA, USA) are two major NGS platforms in PGT-A. Each system uses discrepant library preparation protocols, sequencing principles, and commercial analysis software. The WGA procedures and library preparation in the Ion S5 system are combined, while they are separate in the Miseq system. The Ion S5 system conducts emulsion PCR amplification for library templating using the Ion Chef automatic machine followed with hydrogen ion-detecting sequencing; and the Miseq system employs parallel bridged amplification for optics-based sequencing^18^. Of the throughput, the Ion S5 system can accommodate 16 to 96 samples per run depending on the chips applied, while 24 samples per run as the maximum in the commercial Veriseq PGS kit package on Miseq system. In terms of data analysis, the sequences generated by the Ion S5 and Miseq undergo their own commercialized quality assurance metrics, and then are interpreted using the Ion Reporter software (Thermo Fisher Scientific), and the BlueFuse Multi software (Illumina), respectively. The Ion Reporter automatically achieves aneuploid calling, which can be tuned by a customized analysis workflow; while the BlueFuse Multi requires the operator to conduct manual and observational identification.

Since the Ion S5 and Miseq systems are very different for the identification approach, it is intriguing to compare the ability of ploidy detection between the two NGS systems in PGT-A. In this study, karyotypically defined cell lines were mixed to evaluate the sensitivity and specificity. Then the clinical trophoblast samples were utilized for calculating the concordance per embryo and per chromosome pair.

## Materials and methods

### Study design

This study was approved by the Ethics Review Committee of National Taiwan University Hospital. In the first phase, we employed a mixing experiment with five karyotypically defined cell lines to compare the sensitivity and specificity between the Ion S5 system (Thermo Fisher Scientific) and Miseq system (Illumina). In the second phase, a total of 107 clinical TE biopsies obtained from 40 patients with IVF/PGT-A program underwent two different NGS workflows. The sequencing results were assessed in a double-blinded manner using the Ion Reporter software (Thermo Fisher Scientific) and BlueFuse Multi (Illumina), applying the same calling criteria.

### Cell lines

In order to develop multiple levels of whole and segmental chromosome mosaicism, five cell lines with karyotypically defined ploidy were utilized in the serial mixing experiment. The self-developed amniotic stem cell lines AF01 (46 XX) and AF02 (47 XY, trisomy 21) were kindly provided by the Bioresource Collection and Research Center, Hsinchu, Taiwan^19^. Three cell lines were purchased from the Coriell Cell Repository (Camden, NJ, USA): GM14131 (46 XX,del(5)(p15.1).ish del(5)(p15.33p15.1) (D5S23-). arr 5p15.33p15.1 (68,519–16,362,247) × 1), GM22601 (46 XY,del(4)(p15.2).arr 4p16.3p15.2 (55,665–25,591,051) × 1), AG12070 (47 XX, trisomy 13). The karyotypes of all cell lines were previously identified by the providers. Before utilization, the cell lines were processed according to the suppliers’ recommendation for thawing and passage.

### Internal validation of mosaicism and segmental aneuploidy using cell lines

To compare the sensitivity and specificity of both systems, the karyotypically defined cell lines were utilized for simulating mosaic samples as our previous article^20^. Five to ten individual cells with aneuploid or diploid karyotypes were mixed using glass pipettes at eight specific ratios, as follows: 0% (0:5), 10% (1:9), 20% (1:4), 40% (2:3), 50% (3:3), 60% (3:2), 80% (4:1), and 100% (5:0) (Supplementary Table I). The ratio of 0% aneuploidy was tested for 12 replicates and the other ratios in triplicate. Four validation models were established via serial mixing the diploid cells with trisomy 13, trisomy 21, a 25.5 Mb segmental deletion on chromosome 4 (Wolf-Hirschhorn syndrome), and a 16.2 Mb segmental deletion on chromosome 5 (Cri-du chat syndrome). The samples of mixed cell lines underwent the amplification, library preparation, and sequencing on both NGS platforms. Based on the four models, the sensitivity and specificity could be evaluated, and the common calling criteria for clinical samples between two platforms were furtherly developed. The minimum for segmental aneuploidy was set as 15 Mb. The thresholds of diploid/aneuploid mosaicism were defined as high rate (50%-80% aneuploidy) and low rate (20%-50% aneuploidy), respectively. Euploid was defined as under 20% aneuploidy; and aneuploid was defined as exceeding 80% aneuploidy.

### Clinical subjects

All couples involved in the study were initially counselled by the reproductive consultants. A complete explanation of the IVF/PGT-A process, including published values in terms of the sensitivity and specificity of the Ion S5 and Miseq, the in-house percentage of failed amplification, the inconclusive and aneuploid rate, were provided by the conducting laboratory for their consideration. Each enrolled couple signed the consent form for the study, which was previously approved by the Institutional Review Board of National Taiwan University, before entering the personalized controlled ovarian stimulation program^21^. Informed consent was obtained from all the participants, and all methods were performed in accordance with the relevant guidelines.

Retrieved metaphase II (MII) oocytes were fertilized by intracytoplasmic sperm injection (ICSI) and cultured to the blastocyst stage. Once the inner cell mass (ICM) of the blastocyst was graded above B according to the Gardner and Schoolcraft system^22^ and distinctive cellular TE was evident, biopsy would be performed using pipetting shearing (Origio, Måløv, Denmark).

The biopsied fractions were washed twice in sterile 1X phosphate-buffered saline (PBS) (Cell Signaling Technologies, Danvers, MA, USA) containing 1% (*w/v*) polyvinylpyrrolidone (PVP; Sigma, St. Louis, MO, USA). Then they were gently transferred into a 0.2-mL PCR tube containing 2.5 μL of PBS/PVP solution and stored at − 20 °C.

### Whole genome amplification and library preparation

Both the samples of mixed cell lines and clinical biopsies were thawed, lysed, and randomly fragmented using the extraction and pre-amplification master mix of the Ion SingleSeq kit (Thermo Fisher Scientific). Of samples of mixed cell lines, different whole-genome amplification procedures of individual platforms were carried out in the duplicated samples. Of a single clinical biopsy, each sample must be separated at preamp stage for the different procedures. A total volume of 15 μL fragmented products derived from extracted clinical samples were separated into two aliquots: 7.5 μL for the WGA plus library preparation combined procedure on the Ion S5 system, and the other 7.5 μL for the separate WGA and library preparation procedures on the Miseq system.

To prepare the library for the Ion S5 system, the individual barcodes and amplification master mix of the Ion SingleSeq kit was added to the pre-amplified products. Then a PCR program for both WGA and barcode ligation was performed. The library amplicon was pooled and purified using AMPure XP beads (Beckman Coulter, Pasadena, CA, USA). Then they were quantified using the high-sensitivity (HS) Assay Kit (Qubit, Life Technologies, Waltham, MA, USA), and diluted for templating on the Ion Chef automatic machine (Thermo Fisher Scientific). The templated chip was sequenced using the Ion S5 (Ion ReproSeq PGS Kits-Ion S5 System User Guide).

To prepare the library for the Miseq system, the pre-amplified products were subjected to WGA using the amplification master mix of the Sureplex Amplification kit (Illumina). The amplified products were quantified using the high-sensitivity (HS) Assay Kit (Qubit), and then diluted for preparing the library. The amplicons underwent tagmentation, index ligation, purification by AMPure XP beads, normalization, and eventually they were pooled for the Miseq sequencing (VeriSeq PGS Library Prep Reference Guide).

### NGS and CNV analysis

Data generated by the Ion S5 system was subjected to align to the human reference genome, and went through quality assurance metrics to remove low quality and duplicate reads using Torrent Suite (Thermo Fisher Scientific). Then the available reads were analyzed using Ion Reporter software (Thermo Fisher Scientific) to calculate CNV. The length of a single tile was set as 1 Mb corresponding with the default unit length in the BlueFuse Multi software (lllumina). Aneuploid calling was automatically accomplished by the Ion Reporter with a customized analysis workflow followed by a self-proprietary program for additional tuning. This additional program would filter the noise-like signals out according to the clinical calling criteria applied, which were established from the aforementioned cell line models.

Data generated by the Miseq system was processed and analyzed using the BlueFuse Multi software. Similar but not totally identical, the reads went through a series of quality assurance metrics. To calculate CNV, every aligned read count was assigned to the bin unit with the default length as 1 Mb. Aneuploid calling was conducted manually by well-trained technicians using BlueFuse Multi according to individual observation on the deviation from the default line as copy number two.

### Assessment of sensitivity and specificity

The sensitivity and specificity were calculated using the karyotypically defined cell line mixtures with eight aneuploid ratios: 0%, 10%, 20%, 40%, 50%, 60%, 80%, 100%. Sensitivity was defined as the number of tested samples containing aneuploid cells with positive aneuploid calls divided by the total number of tested samples containing aneuploid cells. Specificity was defined as the number of tested samples containing merely diploid cells without positive aneuploid calls divided by the total number of tested samples containing merely diploid cells.

### Concordance analysis in parallel comparison

Concordance was calculated as per embryo and per chromosome. First, we analyzed the concordance between two systems based on ploidy conclusions for the embryos (euploid and aneuploid). Second, we analyzed the concordance for individual chromosomes (diploid and aneuploid). The same chromosomes with different aneuploid percentages on each NGS system would be counted as concordant, since the aneuploid percentages could be affected by several objective issues, such as the efficiency of WGA and masking of the data processing procedure.

### Internal validation of concordance in separated preamp products using cell lines

The same preamp master mix was used by both library preparation methods in clinical biopsied samples, biasing the results towards the concordance. The errors introduced by the preamp would appear as concordant despite discordance with the actual karyotype. Therefore, we performed randomizing and blinding experiments using three replicate aliquots of 5–10 cells each from five different cell lines with known karyotypes. The procedures of experiments for these cell line samples were totally the same as clinical biopsied samples. Concordances between results of the two platforms and of the original karyotypes were calculated respectively.

### Statistical analysis

The count data  were displayed as percentages, and continuous data as averages and standard deviations (SD). Groups were compared using the Chi-square or Fisher’s exact test. Significant differences were defined as a *p* value less than 0.05. All analyses were conducted using GraphPad software (Prism, GraphPad Software, La Jolla, CA, USA).

## Results

### Patient profiles

Forty couples undergoing IVF/PGT-A were enrolled in this study (mean maternal age: 37.2 years, SD: 4.3 years), including 32 couples using their own oocytes (mean maternal age: 37.2 years, SD: 4.1 years) and 8 couples using donated oocytes (mean donor age: 23.8 years, SD: 2.0 years). In terms of the indications, 20 couples had advanced maternal age (> 36 years) (50%); 6 couples had severe male factors (15%); 6 couples had a history of repeated implantation failure (15%); and 8 couples using donated oocyte (20%) would like to undergo PGT-A for single embryo transfer (SET). Detailed number of retrieved MII, normally fertilized oocytes (two pronuclei, 2PN), derived blastocysts for biopsy, and biopsied samples for NGS testing are displayed in Table 1. One hundred and eight blastocysts were biopsied. One biopsy failed to be amplified (0.9%, 1/108), and a total of 107 clinical biopsies were subjected to the NGS analysis.

**Table 1:** Patient profile.

Number of enrolled couples	40
Mean maternal age (total)	37.2 ± 4.3 (*n* = 40)
IVF with their own oocytes	37.2 ± 4.1 (*n* = 32)
IVF with donated oocytes	23.8 ± 2.0 (*n* = 8)
Number of IVF/PGT-A cycles	46
Indications	
Advanced maternal age (> 36 years)	20 (50%)
Severe male factors	6 (15%)
Repeated implantation failure	6 (15%)
Oocyte recipient for SET	8 (20%)
Number of retrieved oocytes	589
Number of metaphase II oocyte (MII)	514
Number of fertilized oocytes (2PN)	386
Number of derived blastocysts	219
Number of biopsied blastocysts	108
Number of biopsies screened by NGS	107
Number of biopsies that failed to be amplified	1

2PN, 2 pronuclei; NGS, next-generation sequencing; SET, single embryo transfer.

### Assessment of sensitivity and specificity

**Figure 1:**
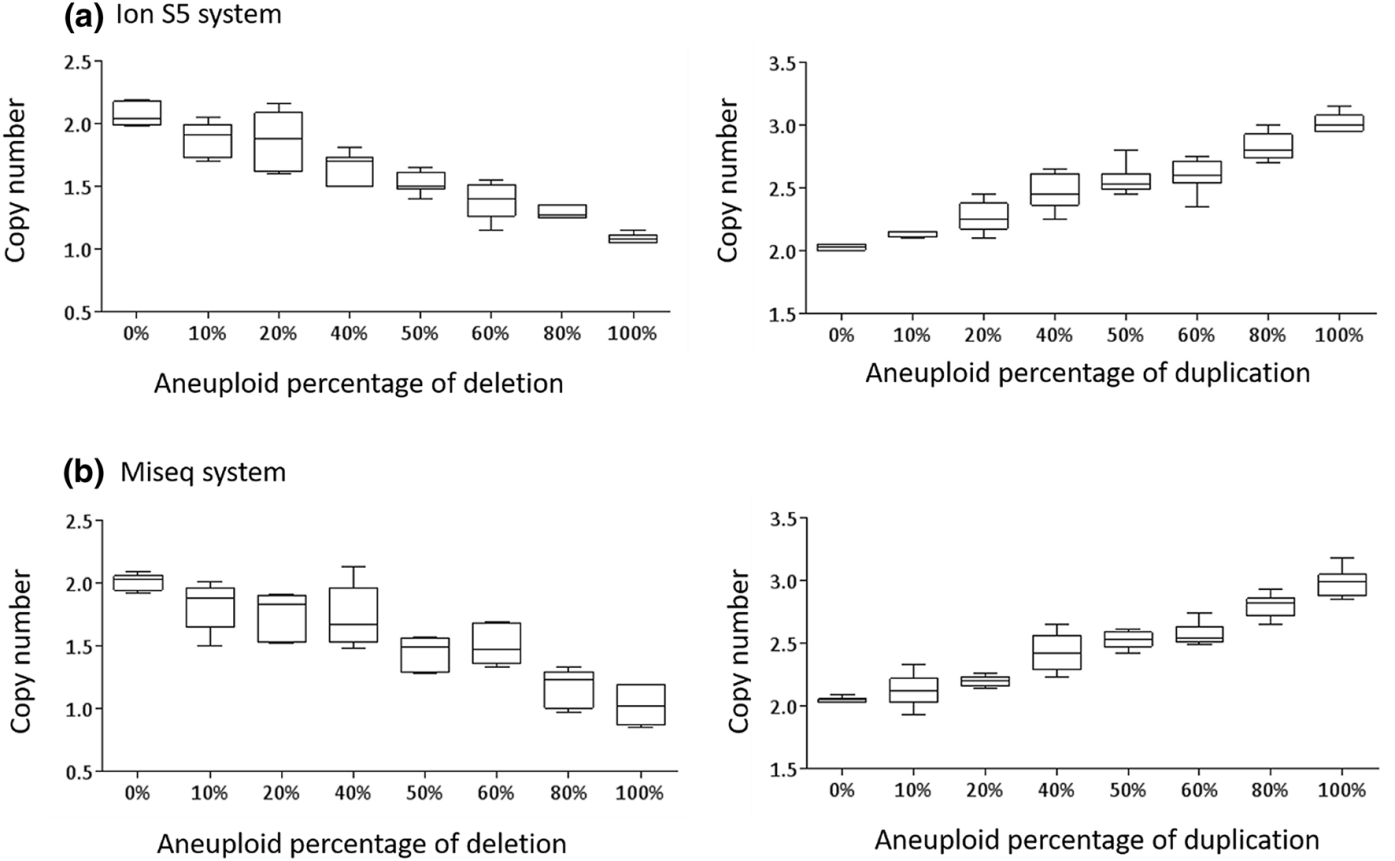
Cell lines are mixed to create multiple levels of aneuploidy. The calculated copy number at the affected aneuploid region displayed correlation with the aneuploid percentage using automatic identification via the Ion Reporter on the Ion S5 system (**1a**), and using manual identification via the BlueFuse Multi on the Miseq system (**1b**). As the number of aneuploid cells in the mixtures increases, the copy number of the regions with segmental deletion or whole-chromosome duplication decreases or increases on both the NGS systems.

Four karyotypically defined aneuploid cell lines were individually mixed with a diploid cell line to simulate mosaic samples with different types and levels of aneuploidy: 0%, 10%, 20%, 40%, 50%, 60%, 80%, and 100%. Figure 1a,b displayed correlation between the aneuploid percentage generated by the mixing experiment and the calculated copy numbers of the affected aneuploid regions determined by the Ion S5 system (based on automatic identification using the Ion Reporter), and by the Miseq system (based on manual identification using the BlueFuse Multi). Both NGS systems reliably distinguished chromosomal CNV of mixtures with at least 10% aneuploidy from karyotypically normal samples ([Ion S5] whole-chromosomal duplication: 2.14 vs. 2.05, *p* value = 0.009, segmental deletion: 1.88 vs. 2.05, *p* value = 0.003; [Miseq] whole-chromosomal duplication: 2.12 vs. 2.03, *p* value = 0.047, segmental deletion: 1.82 vs. 2.03, *p* value = 0.002). Since detection of segmental deletion was more challenging than that of whole-chromosome duplication, the standard deviation displayed a wider range of variation in the box plots.

**Figure 2:**
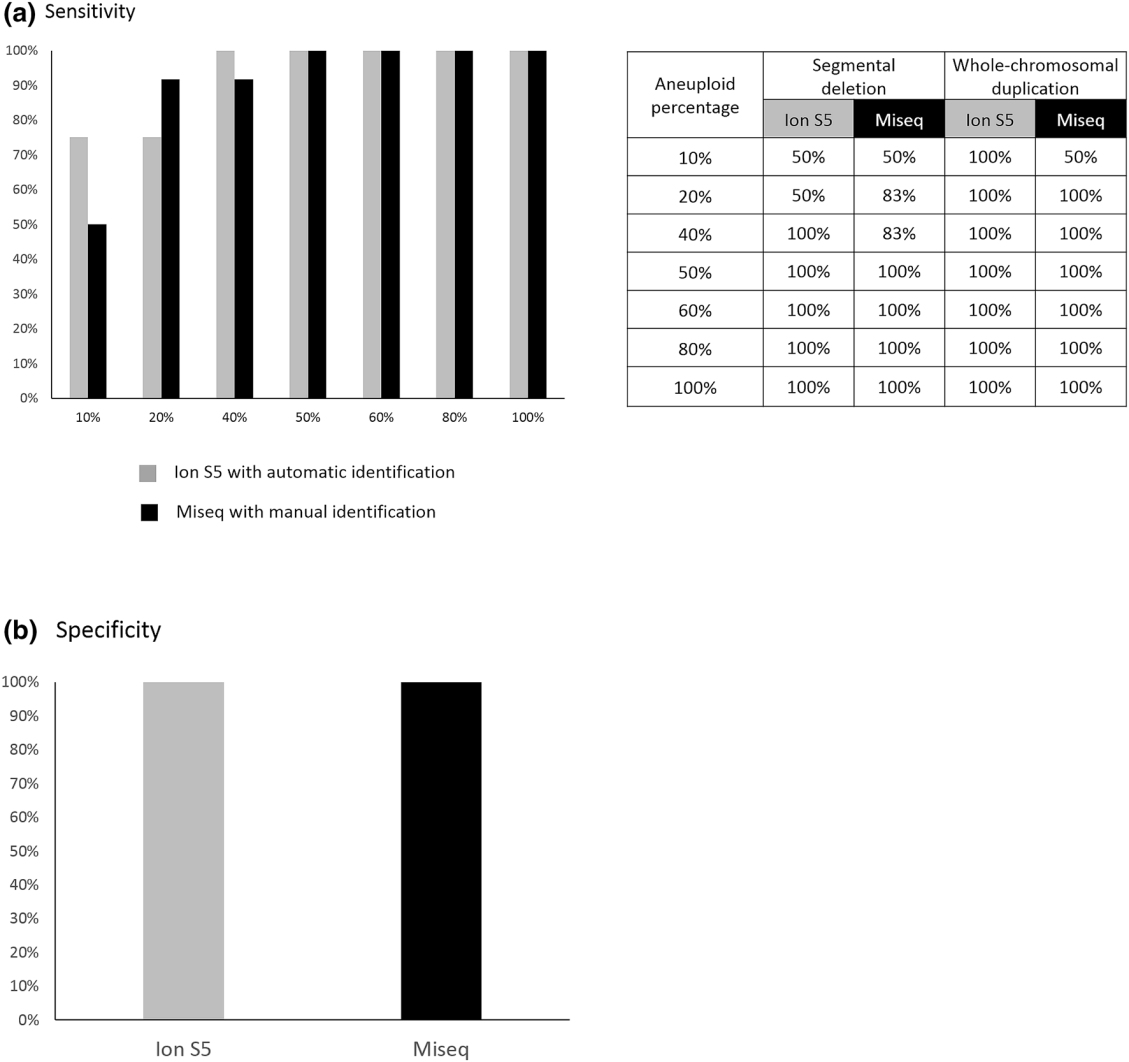
Overall sensitivity of the Ion S5 and Miseq at different aneuploid levels are displayed in the bar chart, and the table lists individual sensitivity for segmental deletion and whole chromosomal duplication (**2a**). Overall specificity of the Ion S5 and Miseq are shown (**2b**). Both the sensitivity and specificity are not significantly different between the two systems.

The overall sensitivity of the two NGS systems at different aneuploid percentages were displayed in the bar chart of Fig. 2a. The individual sensitivity for segmental deletion and for whole chromosomal duplication was displayed in the table of Fig. 2a. No significant difference was reached in the sensitivity: [Ion S5] = 93% vs. [Miseq] = 90%, *p* = 0.78. The overall specificity for the Ion S5 and Miseq were both 100% (12/12) (Fig. 2b), and no significant difference was observed, either (*p* value = 1.00).

### Concordance per embryo

The concordance calculated by the ploidy of embryos between the two NGS systems is presented in Table 2. A total of 107 samples were subjected to sequencing. In chromosomal CNV analysis, three samples displayed chaotic patterns that the ploidy could not be interpreted (2.8%, 3/107). Ploidy was classified as euploid (below 20% aneuploidy), low-rate mosaic (20–50% aneuploidy), high-rate mosaic (50–80% aneuploidy), and aneuploid (exceeding 80% aneuploidy). Concordance was calculated as the number of samples identified as euploid or mosaic/aneuploid on the both two systems divided by the total number of samples with conclusive results. Concordant results were obtained for a total of 103 samples, and thus the concordance rate per embryo was 99.04% (103/104).

**Table 2:** Concordance analysis per embryo.

	Ion S5	Miseq
Number of embryos tested	107	107
Number of embryos screened by NGS	107	107
Number of embryos with conclusive result ^a^	104	104
Number of embryos with inconclusive result	3	3
Number of embryos with aneuploid calling ^b^	54	55
Ploidy conclusion		
Euploid	50 (48.0%)	49 (47.1%)
Low-rate mosaic (20–50% aneuploidy)	6 (5.8%)	11 (10.6%)
High-rate mosaic (50–80% aneuploidy)	8 (7.7%)	9 (8.7%)
Aneuploid	40 (38.5%)	35 (33.6%)
Single-chromosome aneuploidy	28	24
Double-chromosome aneuploidy	5	3
Multiple aneuploidy ^c^	7	8
Number of concordant embryos ^d^	103	
Number of non-concordant embryos ^e^	1	
Concordance per embryo	99.04%	

^a^It excludes embryos with chaotic patterns that the ploidy could not be interpreted.

^b^It includes embryos with mosaicism and aneuploidy.

^c^It includes embryos with more than two aneuploid chromosome pairs.

^d^It includes embryos identified as euploid or mosaic/aneuploid on the both two NGS systems.

^e^Embryos identified as euploid on only one NGS system, and as mosaic or aneuploid on the other system.

### Concordance per chromosome pair

Furthermore, concordance per chromosome pair between the two NGS systems was calculated (Table 3). A total of 2392 chromosome pairs were assessed (52 male embryos and 52 female embryos). One hundred and fifteen chromosome pairs exhibited chaotic mosaicism (2 male embryos and 3 female embryos), and the individual chromosomes affected could not be clearly identified (4.8%, 115/2392). Thus, the remaining 2277 chromosome pairs were categorized as diploid, low-rate mosaic, high-rate mosaic, or aneuploid. Concordance was calculated as the number of chromosome pairs identified as diploid or mosaic/aneuploid on the both systems divided by the total number of chromosome pairs with conclusive results. The same mosaic or aneuploid chromosomes with different aneuploid percentages in a particular sample between the two systems were also counted as concordant. Concordant results were obtained for a total of 2265 chromosome pairs, and thus the concordance rate per chromosome pair was 99.47% (2265/2277).

**Table 3:** Concordance analysis per chromosome pair.

	Ion S5	Miseq
Total chromosome pairs	2392	2392
Chromosomes with conclusive result ^a^	2277	2277
Chromosomes with inconclusive result	115	115
Chromosomes with aneuploid calling ^b^	107	95
Chromosome category		
Diploid	2170 (95.3%)	2182 (95.8%)
Low-rate mosaic (20–50%)	27 (1.2%)	22 (1.0%)
High-rate mosaic (50–80%)	15 (0.7%)	15 (0.7%)
Aneuploid	65 (2.8%)	58 (2.5%)
Overall concordant chromosome pairs ^c^	2265	
Overall non-concordant chromosome pairs ^d^	12	
Concordance rate per chromosome pair	99.47%	

^a^It excludes embryos with chaotic mosaicism, in which the individual chromosomes affected could not be distinguished.

^b^It includes individual chromosome pairs with mosaicism or aneuploidy.

^c^Chromosome pairs identified as diploid or mosaic/aneuploid on the both two NGS systems. Mosaic/aneuploid chromosome pairs with different aneuploid percentages on each system are also included.

^d^Chromosome pairs identified as diploid on the only one system, and as mosaic/aneuploid on the other system.

### Internal validation of concordance in separated preamp products using cell lines

Since the same preamp master mix was used by both library preparation methods in the clinical biopsies, the results may be biased towards the concordance. Any errors derived during the preamp stage would mask the results as concordant despite discordance with their actual karyotype. This issue was addressed by the internal validation of concordance in separated preamp products using karyotypically defined cell lines. Through randomizing and blinding the separated preamp aliquots of initial 5–10 cells each from five different cell lines in three replicates, the concordance between results of the two platforms was 100%, and the concordance to the original karyotype was 93.33%, respectively (Supplementary Table II).

## Discussion

This study evaluated the sensitivity, specificity, and concordance between automatic identification using the Ion S5 system and manual identification using the Miseq system. In the first phase, we compared the sensitivity and specificity of the two systems via karyotypically defined cell line mixtures. In the second phase, we calculated the concordance between the two systems in 107 clinical trophoblast biopsies. The sensitivity and specificity of both two systems were comparable. The concordance per embryo and per chromosome pair were high using the same calling criteria.

NGS technology has been proven to be consistent with many other CCS platforms used in the PGT-A^10,15,23,24^. In early assessments of NGS for PGT-A, the concordance between NGS systems and 24-chromosome aCGH was assessed^10,23^. Accordingly, the sensitivity and specificity of NGS were ultimately high, and the broader dynamic range of CNV status generated by the NGS interpretation software simplified the identification of chromosome ploidy. Subsequently, investigators studied segmental or mosaic aneuploidy using NGS and validated these observations through a third platform, such as FISH or SNP arrays^3,13^. These articles demonstrated that segmental aneuploidy and diploid/aneuploid mosaicism could be identified using NGS, but that not every variation observed was reliable^17^. The WGA artifacts, the algorithms selected for calculation, or the approach of identification can lead to false positiveness^16^. The present study focused on two distinctive identification approaches to evaluate automatic calling using the Ion Reporter software in the Ion S5 system and manual calling using the BlueFuse Multi software in the Miseq system. Though the WGA of two systems were both based on the modified Rubicon PicoPLEX kit (Takara Bio, Kyoto, Japan), their procedures were different in the library preparation. Thus, we separated the pre-amplified products into two aliquots for the parallel comparison. Although it reduced the initial amount of DNA, the performance of libraries could be independently evaluated on each system.

**Figure 3:**
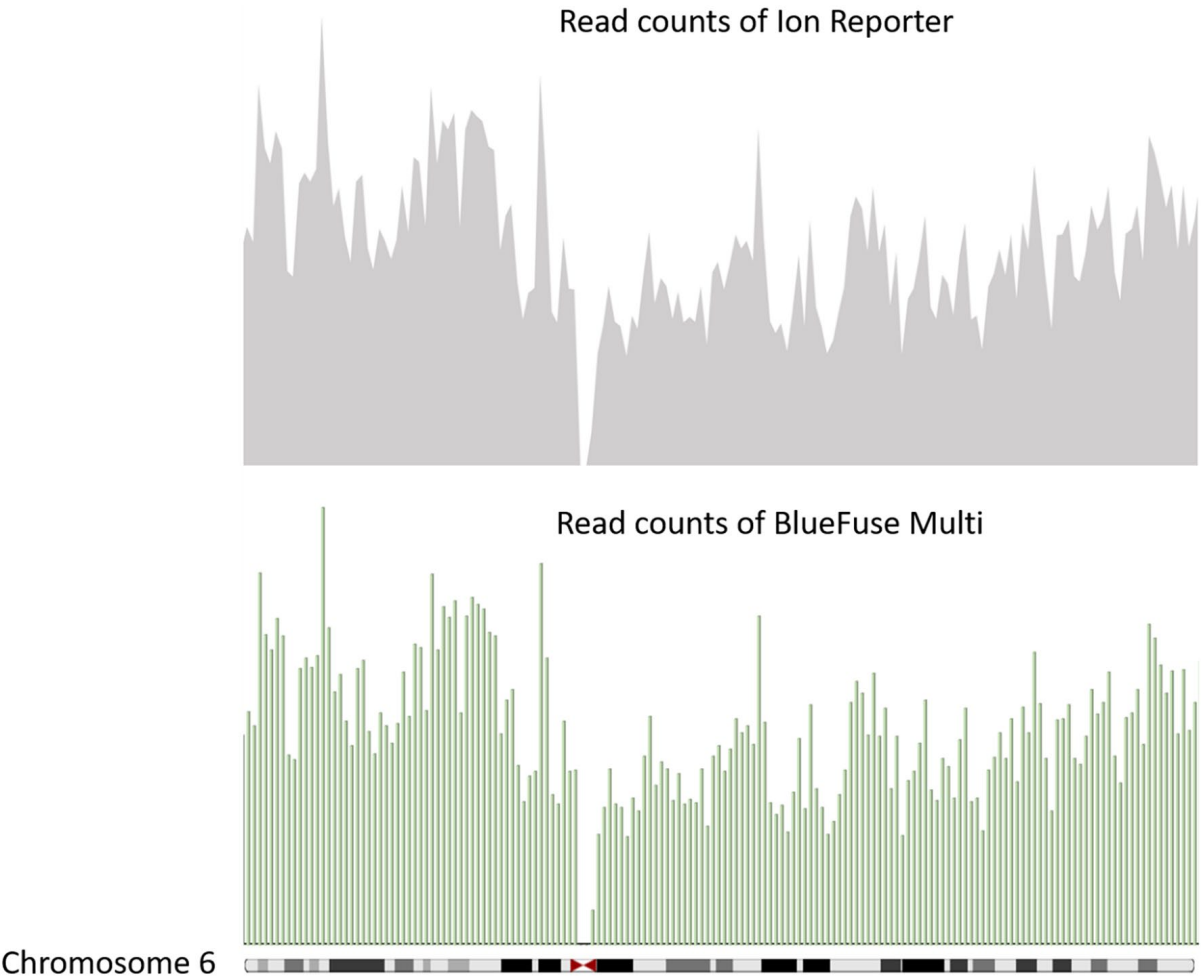
Distribution of read counts aligned within unit intervals (set as 1 Mb) generated by the Ion Reporter software (upper chart), and by the BlueFuse Multi software (lower chart) on chromosome 6. Though the original sequencing lengths are different on each system, the coverage on chromosome 6 displays a similar pattern. The diagram is created using pysam (Release 0.15.0).

Of the sequencing with default setting, the individual sequence length spanned between 100 to 150 bp on the Ion S5, while the Miseq generates uniform 36 bp sequences. Although the sequence lengths are different, the distribution of read counts aligned within unit intervals (set as 1 Mb) across a particular region displayed almost the same pattern between the two systems (Fig. 3). Additionally, their own quality metrics and default setting could be fundamentally similar but not totally identical, due to the specific sequencing principles underlying each system^16^ (Supplementary Table III). Of the CNV region assessment, the Ion Reporter applied a hidden Markov model (HMM) to predict CNV and whole number ploidy status, while the BlueFuse Multi used its own algorithm. Of the measure of background noise in individual samples, the Ion Reporter displayed the median of the absolute values of all pairwise differences (MAPD), while the BlueFuse Multi reviewed the derivative log_2_ ratio (DLR) for the spread of the difference in CNV between all bins within a chromosome (Detection of aneuploidy in a single cell using the Ion ReproSeq PGS View Kit, Application Note, Thermo Fisher Scientific; BlueFuse Multi v4.5, Software Guide, Illumina).

Common calling criteria were applied in the parallel comparison of this study, and it was determined by the former four cell line models. Eventually, high concordance in the clinical samples was obtained between the automatic and manual identifications. However, some differences still existed between the two approaches though common criteria were used. First, the Ion Reporter provided a tunable analysis workflow followed with an automatic identification under this frame, and the BlueFuse Multi allowed operators to make manual calling based upon its own default settings, which are unchangeable (Ion Reporter™ 5.10 User Guide, Thermo Scientific Fisher; BlueFuse Multi v4.5, Software Guide, Illumina). Therefore, some parameters were unable to be completely synchronized between the two software, such as the transition penalty, which represents the sensitivity of different ploidy status between two adjacent data points. Second, manual intervention is not required during automatic identification of the Ion Reporter, while it is necessary for the BlueFuse Multi when the technician observes a deviation from the default line representing copy number two. In some unknown samples with ambiguous patterns, both the masking of automatic identification and subjective conclusions made by manual identification may happen without validation using a second methodology.

In general, batch-to-batch automatic identification was a faster and more standardized approach, but sometimes less flexible in the individual sample. Particularly, the major advantage of automatic workflow provided by the Ion S5 is reduction in manipulation time, reporting time, and thus turnaround time. Of manipulation time, the Ion S5 system combines the WGA and library procedures together, and leaves the remaining steps for the Ion Chef automatic machine. In contrast, the Miseq system takes nearly twice the manipulation time for separate procedures of WGA and library preparation. In terms of reporting time, the automatic identification of Ion S5 system quickly accomplishes typical ploidy calling in a batch, though additional manual rechecks could be required for some ambiguous results; whereas, the manual identification of Miseq system requires individual checking for each sample, and thus needs longer time.

Conclusively, it is the first study to compare the automatic and manual identifications of the Ion S5 and Miseq NGS systems for PGT-A. The sensitivity and specificity of both systems were comparable, while the concordance in the clinical samples was high. The automatic identification provides a faster and more standardized approach, and thus represents a good option for the laboratories with high throughputs.

## Supplementary Information

Supplementary Information 1.

Supplementary Information 2.

Supplementary Information 3.
