# Supplementary material for: High concordance in preimplantation genetic testing for aneuploidy between automatic identification via Ion S5 and manual identification via Miseq
Source: Sci Rep. 2021 Sep 23;11:18931. doi: 10.1038/s41598-021-98318-9 (PMC8460708; doi:10.1038/s41598-021-98318-9)
Supplement: Supplementary file 1 — Supplementary Information 1. [file 41598_2021_98318_MOESM1_ESM.docx]

| **Supplementary Table I. Internal validation of mosaicism and segmental aneuploidy using cell lines** | | | | | |
| --- | --- | --- | --- | --- | --- |
| Karyotype | Aneuploid cell no. | Diploid cell no. | Aneuploid percentage | Miseq | Ion S5 |
|  |  |  |  | Mean copy number (SD) | Mean copy number (SD) |
| 46, XX | 0 | 5 | 0 | 2.04 (0.01) | 2.00 (0.00) |
| 47, XY, +13 | 1 | 9 | 10 | 2.11 (0.20) | 2.14 (0.02) |
| 47, XY, +13 | 1 | 4 | 20 | 2.17 (0.04) | 2.27 (0.08) |
| 47, XY, +13 | 2 | 3 | 40 | 2.55 (0.09) | 2.57 (0.10) |
| 47, XY, +13 | 3 | 3 | 50 | 2.57 (0.04) | 2.52 (0.03) |
| 47, XY, +13 | 3 | 2 | 60 | 2.61 (0.13) | 2.52 (0.14) |
| 47, XY, +13 | 4 | 1 | 80 | 2.83 (0.10) | 2.77 (0.06) |
| 47, XY, +13 | 5 | 0 | 100 | 2.91 (0.08) | 2.97 (0.03) |
| 46, XX | 0 | 5 | 0 | 2.05 (0.03) | 2.05 (0.00) |
| 47, XX, +21 | 1 | 9 | 10 | 2.13 (0.06) | 2.13 (0.03) |
| 47, XX, +21 | 1 | 4 | 20 | 2.23 (0.03) | 2.27 (0.18) |
| 47, XX, +21 | 2 | 3 | 40 | 2.30 (0.07) | 2.37 (0.10) |
| 47, XX, +21 | 3 | 3 | 50 | 2.48 (0.06) | 2.60 (0.18) |
| 47, XX, +21 | 3 | 2 | 60 | 2.53 (0.02) | 2.68 (0.08) |
| 47, XX, +21 | 4 | 1 | 80 | 2.76 (0.10) | 2.88 (0.13) |
| 47, XX, +21 | 5 | 0 | 100 | 3.05 (0.11) | 3.07 (0.08) |
| 46, XX | 0 | 5 | 0 | 2.04 (0.01) | 2.09 (0.10) |
| 46, XY, del(4)(p16.3p15.2) | 1 | 9 | 10 | 1.93 (0.08) | 1.91 (0.18) |
| 46, XY, del(4)(p16.3p15.2) | 1 | 4 | 20 | 1.85 (0.09) | 1.86 (0.23) |
| 46, XY, del(4)(p16.3p15.2) | 2 | 3 | 40 | 1.58 (0.12) | 1.67 (0.16) |
| 46, XY, del(4)(p16.3p15.2) | 3 | 3 | 50 | 1.42 (0.13) | 1.58 (0.08) |
| 46, XY, del(4)(p16.3p15.2) | 3 | 2 | 60 | 1.46 (0.19) | 1.48 (0.08) |
| 46, XY, del(4)(p16.3p15.2) | 4 | 1 | 80 | 1.26 (0.07) | 1.32 (0.06) |
| 46, XY, del(4)(p16.3p15.2) | 5 | 0 | 100 | 1.14 (0.09) | 1.07 (0.03) |
| 46, XX | 0 | 5 | 0 | 1.99 (0.09) | 2.05 (0.11) |
| 46, XX, del(5)(p15.33p15.1) | 1 | 9 | 10 | 1.71 (0.22) | 1.86 (0.10) |
| 46, XX, del(5)(p15.33p15.1) | 1 | 4 | 20 | 1.65 (0.22) | 1.88 (0.27) |
| 46, XX, del(5)(p15.33p15.1) | 2 | 3 | 40 | 1.89 (0.25) | 1.63 (0.12) |
| 46, XX, del(5)(p15.33p15.1) | 3 | 3 | 50 | 1.47 (0.16) | 1.47 (0.06) |
| 46, XX, del(5)(p15.33p15.1) | 3 | 2 | 60 | 1.54 (0.15) | 1.28 (0.13) |
| 46, XX, del(5)(p15.33p15.1) | 4 | 1 | 80 | 1.09 (0.17) | 1.27 (0.03) |
| 46, XX, del(5)(p15.33p15.1) | 5 | 0 | 100 | 0.91 (0.09) | 1.10 (0.05) |
