# Supplementary material for: High concordance in preimplantation genetic testing for aneuploidy between automatic identification via Ion S5 and manual identification via Miseq
Source: Sci Rep. 2021 Sep 23;11:18931. doi: 10.1038/s41598-021-98318-9 (PMC8460708; doi:10.1038/s41598-021-98318-9)
Supplement: Supplementary file 2 — Supplementary Information 2. [file 41598_2021_98318_MOESM2_ESM.docx]

**Supplementary Table II. Internal validation of concordance in separated preamp products using cell lines**

| Karyotype | Aneuploid  cell no. | Diploid  cell no. | Aneuploid  percentage  (%) | Copy number of target site in Miseq (triplicate) | | | Copy number of target site in Ion S5 (triplicate) | | |
| --- | --- | --- | --- | --- | --- | --- | --- | --- | --- |
|  |  |  |  | 1 | 2 | 3 | 1 | 2 | 3 |
| 46, XX | 0 | 5 | 0 | 2.05 | 2.03 | 2.05 | 2.05 | 2.05 | 2.05 |
| 47, XY, +13 | 5 | 0 | 100 | 2.97 | 3.00 | 3.18 | 3.00 | 2.95 | 3.00 |
| 47, XX, +21 | 5 | 0 | 100 | 2.85 | 3.00 | 2.89 | 3.05 | 3.10 | 3.00 |
| 46, XY, del(4)(p16.3p15.2) | 5 | 0 | 100 | 1.19 | 1.04 | 1.08 (ch.10 with 22% decrease,  ch.13 with 20% decrease, ch.18 with 23% elevation) | 1.1 | 0.95 | -* |
| 46, XX, del(5)(p15.33p15.1) | 5 | 0 | 100 | 0.87 | 1.02 | 0.85 | 1.05 | 1.1 | 1.15 |
| Concordance  (between platforms) | 100.00% (14/14) | | | | | | | | |
| Concordance  (to original karyotype) | 93.33% (14/15) | | | | | | | | |

* Blank cells indicate that the aliquot was failed to be amplified, and the sample pair would be excluded in the calculation of concordance between platforms.
