# Supplementary material for: High concordance in preimplantation genetic testing for aneuploidy between automatic identification via Ion S5 and manual identification via Miseq
Source: Sci Rep. 2021 Sep 23;11:18931. doi: 10.1038/s41598-021-98318-9 (PMC8460708; doi:10.1038/s41598-021-98318-9)
Supplement: Supplementary file 3 — Supplementary Information 3. [file 41598_2021_98318_MOESM3_ESM.docx]

**Supplementary Table III**. **Differences between Ion Reporter and BlueFuse Multi software**

| Task item | Ion Reporter | BlueFuse Multi |
| --- | --- | --- |
| Interval length | Changeable (2 Mb, 1 Mb, 0.5 Mb) | Default (1 Mb) |
| CNV transition penalty (sensitivity) | Changeable | Default |
| Baseline | Customized training dataset  (euploid dataset provided by individual labs) | Default |
| Length for segmental aneuploidy | Changeable  (within the frame of selected interval length) | Manually changeable  (default: whole chromosome) |
| Threshold for aneuploid % | Changeable  (within the frame of selected ‘enhanced normal ploidy buffer’; default: 20%) | Manually changeable  (default: 50%) |
| CNV prediction | Hidden Markov model (HMM) | None or not release |
| Background noise assessment | Median of the absolute values of all pairwise differences (MAPD) | Derivative log2 ratio (DLR) |
| Tuning method for aneuploid calling | Parameter setting | Manual |
| Execution of identification | Automatic | Manual |
